# Recruitment Strategies and the Retention of Obese Urban Racial/Ethnic Minority Adolescents in Clinical Trials: The FIT Families Project, Michigan, 2010–2014

**DOI:** 10.5888/pcd12.140409

**Published:** 2015-02-19

**Authors:** Kathryn Brogan Hartlieb, Angela J. Jacques-Tiura, Sylvie Naar-King, Deborah A. Ellis, Kai-Lin Catherine Jen, Sharon Marshall

**Affiliations:** Author Affiliations: Angela J. Jacques-Tiura, Sylvie Naar-King, Deborah A. Ellis, Pediatric Prevention Research Center, Wayne State University School of Medicine, Detroit, Michigan; Kai-Lin Catherine Jen, Department of Nutrition and Food Science, Wayne State University, Detroit, Michigan; Sharon Marshall, Children’s Hospital of Michigan, Wayne State University School of Medicine; Detroit, Michigan.

## Abstract

**Introduction:**

The successful recruitment and retention of participants is integral to the translation of research findings. We examined the recruitment and retention rates of racial/ethnic minority adolescents at a center involved in the National Institutes of Health Obesity Research for Behavioral Intervention Trials (ORBIT) initiative by the 3 recruitment strategies used: clinic, informatics, and community.

**Methods:**

During the 9-month study, 186 family dyads, each composed of an obese African American adolescent and a caregiver, enrolled in a 6-month weight-loss intervention, a sequential multiple assignment randomized trial. We compared recruitment and retention rates by recruitment strategy and examined whether recruitment strategy was related to dyad baseline characteristics.

**Results:**

Of the 186 enrolled families, 110 (59.1%) were recruited through clinics, 53 (28.5%) through informatics, and 23 (12.4%) through community. Of those recruited through community, 40.4% enrolled in the study, compared with 32.7% through clinics and 8.2% through informatics. Active refusal rate was 3%. Of the 1,036 families identified for the study, 402 passively refused to participate: 290 (45.1%) identified through informatics, 17 (29.8%) through community, and 95 (28.3%) through clinics. Recruitment strategy was not related to the age of the adolescent, adolescent comorbidities, body mass index of the adolescent or caregiver, income or education of the caregiver, or retention rates at 3 months, 7 months, or 9 months. Study retention rate was 87.8%.

**Conclusion:**

Using multiple recruitment strategies is beneficial when working with racial/ethnic minority adolescents, and each strategy can yield good retention. Research affiliated with health care systems would benefit from the continued specification, refinement, and dissemination of these strategies.

## Introduction

Excessive body weight is one of the most prevalent medical problems among children and adolescents despite significant attention and funding (1–3). According to 2011–2012 data from the National Health and Nutrition Examination Survey, 39.8% of non-Hispanic black adolescents (aged 12–19) are overweight or obese, compared with 31.2% of non-Hispanic white adolescents (4). Thus the study of weight-loss treatments for adolescents, particularly racial/ethnic minority adolescents, is an important research focus.

The ability to effectively recruit and retain racial/ethnic minority adolescents and their families in research is imperative. Strategies for recruiting and retaining minority research participants emphasize community involvement, convenience of meeting times and locations, and rapport with research staff (5–8). Strategies for recruiting and retaining racial/ethnic minority adolescents for research mirror those recommended for nonminority participants (9–11), with the addition of extensive follow-up (12). Because of the demands of extensive follow-up, successful recruitment and retention of minority adolescents and their families may require substantial time and resources of research staff.

Weight-loss trials among minority adolescents have traditionally used community-based recruitment methods (13,14), such as radio advertisements, or clinic-based methods (15), such as provider referrals. However, clinical informatics —the application of information technology (eg, screening for eligible participants using electronic medical records [EMRs]) — can increase the quality and efficiency of clinic-based methods by incorporating the processes and resources of the biomedical sector (16,17). Informatics, when used in addition to traditional recruitment strategies, can improve enrollment by enhancing identification of and access to participants.

The objectives of this study were to 1) describe the enrollment of obese racial/ethnic minority adolescents in a 6-month weight-loss intervention (FIT Families) using 3 recruitment strategies (clinic, informatics, community), 2) compare the 3-month, 7-month, and 9-month retention rates of the 3 strategies, and 3) identify baseline participant characteristics that may be associated with retention rates.

## Methods

### Study design

Our center, the Wayne State University Pediatric Weight Management Center, as a partner in the National Institutes of Health’s initiative, Obesity Research for Behavioral Intervention Trials (ORBIT), brought together a multidisciplinary research group composed of 1) obesity intervention researchers with extensive experience in adolescent health behavior change, 2) basic behavioral scientists with experience in motivation and learning, 3) registered dietitians and nutritionists with expertise in dietary and weight-loss interventions, and 4) communication scientists who focus on interactions between health providers and families in urban populations. The goal of the center was to develop an adaptive weight-loss treatment for obese African American adolescents. Establishing successful recruitment approaches to allow for maximum retention was a vital component.

FIT Families was a 6-month sequential multiple assignment randomized trial (SMART) (18) focusing on weight loss among obese African American adolescents (19). Recruitment began in November 2010, the first data collection took place in January 2011, and the last data collection took place in March 2014. The goal of using the SMART approach was to develop an evidence-based adaptive intervention that would be evaluated in a subsequent randomized controlled trial (20). Our SMART design had 2 randomization points (Figure). Each study arm included an intervention contact twice per week (except for the maintenance stage, which consisted 1 session per week). Each participating family received $50 for completion of data collection at 3 points (baseline, 7 months, and 9 months) and $10 for completion of data collection at 3 months. Details on the intervention are described elsewhere (19).

**Figure:**
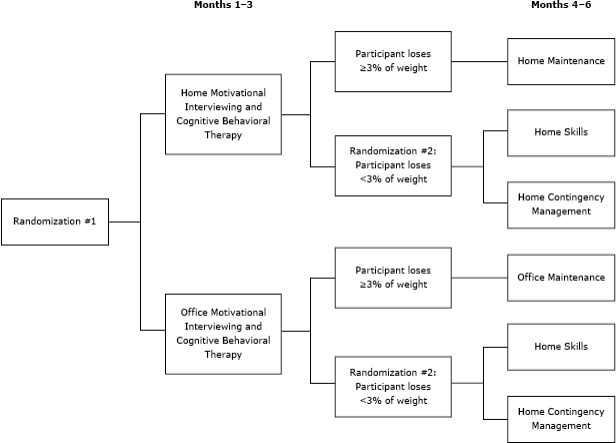
Overview of sequential multiple assignment randomized trial (SMART) design (19) for the FIT Families study, Michigan, 2010–2014. The first randomization took place at baseline and the second at 3 months. The second randomization took place if the adolescent participant did not lose 3% of his or her initial body weight. “Office” refers to the Wayne State University Pediatric Weight Management Center.

### Eligibility criteria

Study inclusion criteria for the adolescent were 1) self-identified as African American, 2) aged from 12 years, 0 months through 16 years, 11 months at time of consent, 3) body mass index (BMI) for age in the 95th percentile or more, 4) resides 30 miles or less from study offices, 5) resides with the primary caregiver, 6) primary caregiver willing to participate in treatment, and 7) English speaking. Adolescents were excluded for the following reasons: 1) obesity was secondary to medication (eg, steroids, antipsychotics) or a chronic health condition (eg, Prader-Willi Syndrome); 2) pregnancy, 3) medical conditions for which weight loss was contraindicated, 4) thought disorder, or 5) serious cognitive impairments. For each adolescent participant, we recruited a primary caregiver so that our primary unit of analysis was a family dyad. Eligibility criteria for the caregiver were being at least 18 years old; being the legal guardian or, if not the legal guardian, having the consent of the legal guardian; and willingness to participate in treatment. Research protocols were approved by the Wayne State University Human Investigation Committee, and investigators reported outcomes every 6 months to a data safety and monitoring board.

### Recruitment strategies

#### Clinics

Clinic-based recruitment took place in a large urban children’s hospital that serves 60,000 children annually. This strategy involved direct collaboration with clinic health care providers. A 15-minute orientation was held with outpatient health care providers from various disciplines (eg, adolescent medicine, ambulatory pediatrics, asthma, diabetes); the session emphasized the importance of study eligibility criteria. Health care providers received a study binder that contained information on eligibility criteria, scripts for introducing the study and talking about it to potential family participants, and brochures to support a 1-minute introduction (including research staff contact information) of the study to families. Families interested in learning more about the study were asked to complete a release-of-information form so that research staff could follow up with further information. Research staff spent approximately 1 hour each week visiting clinics to collect forms and provide a visual reminder of the study to health care providers. A nurse manager was the main contact in clinics with large numbers of rotating residents (eg, ambulatory pediatrics); an attending physician was the main contact in specialty clinics (eg, diabetes). Clinic providers expressed appreciation for the option of offering a weight-management resource to patients and did not request additional compensation. This recruitment strategy allowed the research team to recruit from multiple clinics while making minimal demands on the time of clinical and research staff.

#### Informatics

Unlike younger children, adolescents often do not have medical appointments for regular well-child check-ups (21,22); this lack of regular medical checkups limits researchers’ ability to recruit adolescents through clinics. Therefore, we added the use of informatics as a second recruitment strategy. Although BMI data in the EMRs at some of the clinics offered the potential to identify eligible adolescents, we could not use BMI percentile as a search criterion in the overall hospital EMR system. Instead, we relied on medical billing information in the EMR for data on obesity. Informatics identified adolescents who were seen as inpatients, in outpatient clinics, or in the emergency department for whom medical billing included an *International Classification of Diseases, Ninth Revision* code for obesity, acanthosis nigricans, metabolic syndrome, or type 2 diabetes. A recruitment letter and a study brochure were mailed to potentially eligible families from the hospital’s adolescent medicine division. The letter stated that the family had 2 weeks to opt out of being contacted for the study; otherwise a FIT Families research assistant would telephone to provide more information.

#### Community

Community-based recruitment consisted of free postings (eg, flyers on bulletin boards, brochures) at institutions serving racial/ethnic minority populations and media announcements through the university and hospital. Research assistants offering nutrition and study information attended 5 health fairs at local schools and churches. This recruitment strategy also included referrals through word-of-mouth by enrolled participants to others in the community.

### Screening and enrollment 

Participant screening and enrollment involved a 3-step EMR-based, telephone-based, and home-based process. Research staff spent 1.5 hours per week reviewing EMR data to exclude adolescents who did not meet eligibility criteria. EMR screening often provided eligibility information on adolescent race/ethnicity, height and weight (for calculation of BMI), age, and distance of residence from study offices. Telephone screening was completed with the adolescent’s primary caregiver. The time spent on the telephone with each family recruited through the clinic-based and community-based strategies typically was 20 minutes, whereas research assistants typically spent an additional 5 to 10 minutes on the telephone with each family recruited through the informatics strategy because these families had less knowledge of the study. Research staff initially attempted to contact families twice weekly following a standardized structure of different times (morning, midday, afternoon, evening) on different days of the week and weekend. Brief messages with call-back information were left on machines (when possible) or with people other than the primary caregiver; confidentiality was maintained. When a family could not be reached after 1 month, research staff called the family twice per month. Once a family was reached, the telephone screen entailed a series of questions to establish eligibility. A family was counted as a passive refusals or lost contact if the research staff could not reach it for any reason (eg, telephone messages were disregarded, contact information was incorrect). If eligibility was established by the telephone screen, the research assistant scheduled a home-based screening and consent visit. After research staff confirmed eligibility in person during the home visit, which included a screening for the potential participant’s level of safety in engaging in physical activity, the researcher obtained informed consent from the caregivers and assent from the adolescents. Because of the complex SMART study design, extra attention was paid to explain the 6 intervention arms to study participants (19). Differences between compensation for data collection and intervention visits were also explained. The home visit took 30 to 45 minutes. After providing consent, families completed the 2-hour data collection process; which included questionnaires and anthropometric measurements conducted by the research assistants. For this report, we collected data on the following characteristics: height and weight of the adolescent and the caregiver (to calculate BMI as weight in kg divided by height in meters square); age of the adolescent, any comorbidities of the adolescent, annual income of caregiver, and education of caregiver.

### Retention

To retain families for the data collection at 3 months, 7 months, and 9 months, a reminder postcard was sent to families 4 weeks before data collection began. Telephone calls to schedule data collection appointments started 3 weeks before data collection. If a family was within 1 week of data collection and had not yet confirmed an appointment with the research staff, more intensive efforts began. These included more frequent telephone calls, home visits, or reaching out to the alternate contact people identified by the caregiver during the consent process.

### Staff training

Staff training for recruitment, enrollment, and retention involved role playing and observation to ensure the research staff’s fidelity to protocol. Scripts were developed for research staff to emphasize a person-centered, nondirective communication style using open questions and reflections. Quality assurance checks were conducted every 6 months by a supervisor who observed the screening and consent and assent and data collection processes. Research staff and investigators discussed recruitment and retention efforts during weekly meetings. The equivalent of 2.25 full-time research assistants was employed for 37.5 hours per week during the study period. The 0.25 full-time research assistant helped during home-based data collection.

### Analytic plan

We examined the number and percentage of families recruited from each of the 3 recruitment strategies and used χ^2^ contingency tables to determine whether any strategy was associated with study enrollment or retention at 3 months, 7 months, or 9 months. We used analysis of variance (ANOVA) and Tukey’s honestly significant difference (to account for multiple comparisons) post hoc tests to determine whether any strategy was associated with the age or BMI of the adolescent or BMI of the caregiver (all continuous variables), and we used a χ^2^ test to determine whether any recruitment strategy was associated with the presence of adolescent comorbidities, caregiver education, or caregiver income (categorical variables). We calculated an overall enrollment rate (number of families enrolled divided by the number originally identified), and active refusal rate (number of families who actively refused divided by the number originally identified), a passive refusal rate (number of passive refusals or lost contacts divided by the number originally identified), a modified active refusal rate (number of active refusals divided by the number who passed the EMR screen), and a modified passive refusal rate (number of passive refusals or lost contacts divided by the number who passed the EMR screen).

## Results

### Recruitment

Of the 1,036 families identified through clinics, informatics, and community, only 30 (2.9%) actively refused to participate and all who refused did so during the telephone screening (Table 1). However, 402 families passively refused or were lost contacts. Overall passive refusal rate was related to recruitment strategy (χ^2^_2_ = 28.4, *P* < .001), with a larger percentage of families passively refusing who were identified through informatics (45.1%) than through clinic (28.3%) or community strategies (29.8%). Among families who passed the EMR and other prescreening (n = 679), the modified passive refusal rate was related to recruitment strategy (χ^2^_2_ = 92.5, *P* < .001), with a larger percentage of families passively refusing who were identified through informatics (74.9%) than through clinics (39.1%) or the community (34.7%). Of families who consented to participate (n = 197) but did not enroll, 7.0% were recruited through informatics and 6.0% through clinics; all families recruited through the community consented and enrolled (χ^2^_2_ = 1.6, *P* = .44). Of the 186 enrolled families, 28.5% were identified through informatics, 59.1% through clinics, and 12.4% through the community. Enrollment rate and recruitment strategy were significantly related (χ^2^_2_ = 110.4, *P* < .001). Although the fewest number of families were identified through the community (n = 23), 40.4% enrolled, compared with 8.2% through informatics and 32.7% through clinics.

**Table 1:** Potential Participants in a Weight-Loss Intervention for Obese Racial/Ethnic Minority Adolescents, by Recruitment Strategy, Michigan, 2010–2014

Component	Informatics	Clinics	Community	Total
**Potential participants identified, by strategy, n**	643	336	57	1,036
**Screening of EMRs^a^, n**				
Ineligible individuals	254	68	8	330
Eligible individuals	387	243	49	679
**Other reasons for ineligibility or nonparticipation during EMR screening, n**				
Ineligible because of participation in earlier study^b^	2	25	0	27
Refused after receiving opt-out letter^c^	0	NA	NA	0
**Screening by telephone, n**				
Passive refusal or lost contact	268	73	12	353
Telephone screens completed	119	170	37	326
Active refusal	15	14	1	30
Ineligible	25	16	6	47
Eligible	79	140	30	249
**Screening in the home, n**				
Passive refusal or lost contact	18	15	5	38
Home screens completed	61	125	25	211
Active refusal	0	0	0	0
Ineligible	4	8	2	14
Eligible and consented	57	117	23	197
**Consented but not enrolled, n**	4	7	0	11
**Family enrollment**				
Families enrolled, no. (%^d^)	53 (28.5)	110 (59.1)	23 (12.4)	186 (100)
Overall enrollment rate, %^e^	8.2	32.7	40.4	17.8
Active refusal rate, %^f^	2.3	4.2	1.8	2.9
Passive refusal rate, %^g^	45.1	28.3	29.8	39.8
Modified active refusal rate, %^h^	3.9	5.8	2.0	4.4
Modified passive refusal rate, %^i^	74.9	39.1	34.7	57.6

Abbreviations: EMR, electronic medical record; NA, not applicable.

a Screening of EMRs was done for families identified through the informatics strategy, the clinic strategy, and when applicable, the community strategy.

b People who participated in an earlier study or whose siblings participated in an earlier study were deemed ineligible.

c The opt-out letter was sent only to families identified through the informatics strategy.

d Percentage of families enrolled, by recruitment strategy.

e Number of enrolled families divided by the number originally identified.

f Number of active refusals divided by the number originally identified.

g Number of passive refusals or lost contacts divided by the number originally identified.

h Number of active refusals divided by number deemed eligible by EMR screening.

i Number of passive refusals or lost contacts divided by number deemed eligible by EMR screening.

### Retention

Five families were removed from the study by the principal investigator, 2 because of interventionist error and 3 because of discovery of ineligibility after the study began; 1 family was recruited through informatics, 1 from the community, and 3 recruited from clinics. Thus, 181 families were expected to complete all aspects of the study. No recruitment strategy was related to retention rates at 3 months (χ^2^_2_ = 1.9, *P* = .39), 7 months, (χ^2^_2_ = 1.9, *P* = .38), or 9 months (χ^2^_2_ = 1.8, *P* = .41) (Table 2). Overall study retention was 87.8%.

**Table 2:** Retention Rates in a Weight-Loss Intervention for Obese Racial/Ethnic Minority Adolescents (N = 181), by Recruitment Strategy, Michigan, 2010–2014

Strategy	Baseline, No.	3 Months, No. (%)	7 Months, No. (%)	9 Months, No. (%)
Informatics	52	49 (94.2)	48 (92.3)	48 (92.3)
Clinics	107	95 (88.8)	91 (85.0)	93 (86.9)
Community	22	21 (95.5)	20 (90.9)	18 (81.8)
Overall	181	165 (91.2)	159 (87.8)	159 (87.8)

### Baseline characteristics

Recruitment strategy was not related to the BMI or age of the adolescent, presence of comorbidities, caregiver BMI, caregiver education level, or caregiver income (Table 3).

**Table 3:** Baseline Characteristics of Participants (N = 181) in a Weight-Loss Intervention for Obese Racial/Ethnic Minority Adolescents, Recruitment Strategy, Michigan, 2010–2014^a^

Characteristic	Strategy			Omnibus Test (*P* Value)
	Informatics	Clinics	Community	
BMI of adolescent, kg/m^2^	37.8 (7.7)	37.6 (7.0)	41.5 (8.4)	*F*_2, 178_ = 2.62 (.08)
BMI of caregiver, kg/m^2^	43.5 (11.8)	40.4 (9.3)	37.7 (9.2)	*F*_2, 176_ = 3.46 (.05)
Age of adolescent, y	13.7 (1.2)	13.8 (1.4)	13.4 (1.4)	*F*_2, 178_ = 0.81 (.45)
Percentage of adolescents with at least 1 comorbidity	57.7	49.5	31.8	χ^2^_2_ = 4.14 (.13)
Education level of caregiver^b^	5.4 (1.4)	5.3 (1.3)	5.6 (1.7)	*F*_2, 178_ = 0.47 (.62)
Annual income of caregiver^c^	3.7 (2.1)	3.2 (1.8)	3.4 (2.6)	*F*_2, 175_ = 0.79 (.45)

Abbreviation: BMI, body mass index.

a All values are mean (standard deviation) unless otherwise indicated.

b Response scale for caregiver education level: 1, did not finish elementary school; 2, finished middle school; 3, finished some high school; 4, high school graduate or general educational development (GED); 5, vocational or training school after high school; 6, some college or associate degree; 7, college graduate or baccalaureate degree; 8, master’s or doctoral degree.

c Response scale for caregiver income: 1, <$5,000; 2, $5,000–$11,999; 3, $12,000–$15,999; 4, $16,000–$24,999; 5, $25,000–$34,999; 6, $35,000–$49,999; 7, $50,000–$74,999; 8, $75,000–$99,999; 9, ≥$100,000.

## Discussion

The 3 recruitment strategies used in this study demonstrated processes to identify and promote research participation among obese racial/ethnic minority adolescents and their caregivers. More than half (59.1%) of families were enrolled through the clinic-based strategy. The personal interaction with a medical clinic health care provider may have had a beneficial influence. Qualitative studies have reported positive experiences during clinician-initiated recruitment despite clinicians’ initial misgivings about burdening or overwhelming families (23). A discussion about research opportunities in the context of clinical care can build patient–provider relationships regardless of research participation (24) and can thus be valuable to clinicians, patients, and researchers. Overcoming clinicians’ negative feelings about approaching families for participation in research was achieved in this study through orientation sessions and well-defined recruitment procedures.

The findings of this study also indicate the value of recruitment strategies other than clinic-based recruitment. Although the proportion of families enrolled through the community (12.4%) was the lowest of the 3 recruitment strategies, 40.4% of identified eligible participants enrolled, compared with only one-third of those identified through clinics and less than 10% through informatics. Community-based recruitment of racial/ethnic minority participants fosters trust and is a positive way to reach minority and adolescent populations (5,25). Community-based recruitment was a more passive strategy (eg, health fair conversations) than the other 2 strategies but was an important avenue for linking interested and motivated participants to research.

Despite lacking the advantages of personal interaction, the informatics strategy enrolled more than one-quarter of eligible families that may otherwise have been overlooked; it is a viable approach for health care system–based research. The use of opt-out letters meant that potentially eligible families did not have to proactively indicate an intention to participate. This strategy has been found to shorten recruitment time and double the number of participants compared with an opt-in letter recruitment strategy (26). No families receiving letters in our study opted out of being contacted by research staff. The benefits of the informatics strategy may be even more pronounced during the latter years of recruitment for multiyear clinical trials when clinic and community referrals plateau.

Although our study had few active refusals (3%) across the 3 strategies, we had many passive refusals. We were not able to screen by telephone almost three-fourths of families identified though informatics, compared with about half of families identified through clinics and one-third of families identified through the community. Overall, our findings support previous research. Racial/ethnic minority youths and families tend to participate when reached through appropriate recruitment strategies (27), and multiple strategies are required for success (28).

Because BMI data were not available in the hospital EMR system, we relied on obesity-related diagnoses for the informatics strategy. Clinicians may not submit billing for obesity unless a significant amount of excess weight is present. Therefore, during recruitment, we anticipated being unable to identify some eligible adolescents and possibly introducing bias by identifying adolescents at the upper end of the BMI scale. However, we found no differences among the populations across the 3 methods. Our recruitment strategies allowed for inclusivity of participants across BMI, age, comorbidity status, and socioeconomic status of the study location.

Our retention program targeted several key areas recommended in the literature (5,28). Research staff provided accessible locations for meetings, frequent reminders, and timely incentive payments, and, for the most part, staff was consistent during the study. Overall, our study retention rate (88%) was within the range found in similar studies (29,30) and did not differ across recruitment strategies. 

Our recruitment methods had limitations. Health care providers participating in the clinics did not record the number of families who were approached but refused to participate. Such data could provide important insight into the total amount of time spent by providers and research staff on recruitment. A formal time-allocation study, particularly on screening and initial contact, is warranted. Additional data are being compiled through exit interviews with study participants. An advisory board made up of community members and study participants will also be convened after completion of the study to further refine recruitment strategies.

The 3 recruitment strategies described here — clinic, informatics, and, community — led to the successful inclusion of racial/ethnic minority adolescent participants across numerous baseline characteristics. Study retention strategies were efficacious and may be practical for other research groups affiliated with health care–based systems. Research in behavioral intervention trials would benefit from the continued specification, refinement, and dissemination of these recruitment and retention strategies.
